# Human Umbilical Cord Mesenchymal Stem Cell-Derived Exosomes Improve Ovarian Function and Proliferation of Premature Ovarian Insufficiency by Regulating the Hippo Signaling Pathway

**DOI:** 10.3389/fendo.2021.711902

**Published:** 2021-08-12

**Authors:** Zhongkang Li, Mingle Zhang, Jiahua Zheng, Yanpeng Tian, Huihui Zhang, Yi Tan, Qian Li, Jingkun Zhang, Xianghua Huang

**Affiliations:** ^1^Department of Obstetrics and Gynecology, The Second Hospital of Hebei Medical University, Shijiazhuang, China; ^2^R & D Department, Qilu Cell Therapy Technology Co., Ltd, Jinan, China; ^3^Institute of Immunotherapy, Shandong Yinfeng Life Science Research Institute, Jinan, China

**Keywords:** premature ovarian insufficiency, mesenchymal stem cell, exosome, hippo signaling pathway, reproduction, proliferation

## Abstract

**Background:**

Premature ovarian insufficiency (POI) is associated with severe physical damage and psychological burden on women. Transplantation of exosomes is an encouraging regenerative medicine method, which has the potential for restoring ovarian functions on POI with high efficiency. This study aims at evaluating the therapeutic efficacy of human umbilical cord mesenchymal stem cell-derived exosomes (hUCMSC-Exos) on ovarian dysfunction of POI and the role of Hippo pathway in this exosome-mediated treatment.

**Methods:**

POI mice models were established through intraperitoneal injection of cyclophosphamide. Subsequently, transplantation of hUCMSC-Exos was conducted to administer POI mice. Ovaries and plasma of these mice models were harvested after two weeks of treatment. Ovarian morphology and follicle number were assessed by hematoxylin and eosin staining. Moreover, ELISA was used to detect hormone levels, which are related to ovarian function in serum. To assess the recovery of reproductive ability, we recorded the rate of pregnancy, the amount of offspring, and the time of birth in different groups. To explore the underlying mechanisms of exosome-mediated treatment for ovarian function recovery, the proliferation of ovarian cells *in vivo* was detected by immunohistochemistry and immunofluorescence staining. Additionally, we conducted EdU and CCK-8 assays to assess the proliferative ability of ovarian granulosa cells (GCs) that were cultured *in vitro*. Western blot analysis was conducted to estimate the proteins levels of Hippo- and proliferation-associated molecules *in vivo and in vitro*.

**Results:**

After transplantation of hUCMSC-Exos, the ovarian function-related hormone levels and the number of ovarian follicles returned to nearly normal degrees. Meanwhile, there was a significant improvement in reproductive outcomes after exosomal treatment. Furthermore, the improvement of ovarian function and proliferation was associated with the regulation of Hippo pathway. *In vitro*, co-culture with exosomes significantly elevated the proliferation of ovarian GCs by regulating Hippo pathway. However, the positive effects on the proliferation of GCs were significantly depressed when key Hippo pathway molecule was inhibited.

**Conclusion:**

This study suggested that hUCMSC-Exos promoted ovarian functions and proliferation by regulating the Hippo pathway. Therefore, exosomal transplantation could be a promising and efficient clinical therapy for POI in the near future.

## Introduction

Premature ovarian insufficiency (POI) is a complicated endocrine disease with a worldwide incidence of 1-5% (1, 2). In clinical practice, diagnosis of POI is accepted in women below 40 years who present with amenorrhea for beyond four months, with deficiency of sexual hormones, and with follicle stimulating hormone (FSH) levels in serum above 40 IU/L (3). In most cases, various and diverse factors lead to premature dysfunction or depletion of ovarian follicles. Although etiologic elements include genetic, autoimmune, and iatrogenic factors, the specific etiology is unclear in most cases (4, 5). It is mainly characterized by declining ovarian functions, which were related to amenorrhea, high gonadotropin, and low estrogen levels (6). Occurrence of POI at a young age could lead to severe outcomes on reproductive health and may cause infertility. Additionally, estrogen deprivation is associated with sexual dysfunction, cardiovascular disease, low bone density, and even severe psychological burdens. Although hormone replacement therapy (HRT) is the most conventional clinical treatment option for administering POI patients, it has unsatisfactory efficacy owing to the non-fundamental restoration of ovarian function and various side effects (7).

Mesenchymal stem cell (MSC) is a type of non-hematopoietic stem cell with multidirectional differentiation potential and low immunogenicity (8). Several efforts have been taken to effectively apply MSCs in numerous diseases. Meanwhile, MSC-related therapies have the potential for reversing ovarian aging and dysfunction because of their therapeutic characteristics, including migration ability and paracrine effects (9). Experimental outcomes of various kinds of MSCs in POI are encouraging (10, 11). Among them, human umbilical cord MSCs (hUCMSCs) have been widely used because of easier isolation, lower immunogenicity, and higher proliferative capacity (12). The paracrine effect has been reported to mediate the therapeutic effects of MSCs (13, 14). Specifically, paracrine effectors of their secretome, including non-coding RNAs, cytokines, and growth factors, can be transplanted to target cells in damaged organs or tissues to perform long-term effects. Extracellular vesicles (EVs) are the major paracrine effectors that can transfer bioactive components (15). As a type of EVs, exosomes are essential in cell-to-cell communication by delivering numerous self-carrying proteins and non-coding RNAs (16). In this way, exosomes can boost tissue responses to damage and disease (17). Because of their special structures, exosomes are stable enough *in vivo* compared to other kinds of particles (18). These characteristics enhance their clinical applications for POI. Therefore, this cell-free regenerative medicine provides a new low-risk therapy for POI. Previous studies have reported that exosomes derived from hUCMSC (hUCMSC-Exos) could administer POI by inhibiting the apoptosis of ovarian granulosa cells (GCs) and regulating immune cell responses (19, 20). However, specific and underlying therapeutic mechanisms of exosome-mediated restoration of ovarian functions and folliculogenesis have not been fully understood.

The Hippo pathway is critical in folliculogenesis and ovarian function by regulating follicle activation and survival, and ovarian cells proliferation (21). The key molecules of the Hippo pathway, including Large tumor suppressor 1/2 (LATS1/2), Mammalian Ste20-like kinases 1/2 (MST1/2), Yes-associated protein (YAP), PDZ-binding motif (TAZ), and TEA domain transcription factor (TEAD), play a critical regulatory function in the cytoplasm of GCs (22–24). Meanwhile, ovarian follicular development is associated with elevated YAP, TEAD, and TAZ levels, and suppressed MST1 and LATS levels (22). Taken together, the Hippo pathway is essential in regulating ovarian follicles’ function and activation. However, the role of Hippo pathway in the exosome-mediated treatment for POI has not been fully explored.

MSCs have exhibited significant therapeutic potential for POI at present. However, the therapeutic efficiency of hUCMSC-Exos and underlying molecular mechanisms are far from well elucidated. This investigation was systematically designed to confirm the therapeutic efficiency of exosomal therapy in promoting ovarian function for POI and explore the regulatory role of Hippo pathway in exosome-mediated treatment, which is fundamental for the further conduction of this novel approach for POI.

## Methods

### Identification of hUCMSCs

Third passage hUCMSCs were purchased from Qilu Cell Therapy Technology (Shandong, China) and were cultured in Dulbecco’s modified Eagle’s medium (DMEM; Gibco, US), which was supplemented with 10% exosome-depleted fetal bovine serum (Gibco) and 1% penicillin/streptomycin (Gibco), at cellular incubation conditions (37°C and 5% CO_2_). The MSCs were identified as previously described (25). First, we confirm their morphology by using a light microscope (Imager.D2; ZEISS, Germany). Then, Alizarin Red staining was used for osteogenic identification; and Oil Red O staining was conducted for adipogenic differentiation. Finally, Flow cytometry (FCM; FACSCanto II, BD, US) was used to detect MSC-related surface markers (CD34, CD44, CD45, CD73, CD90, CD105, and HLA-DR; Biolegend, US).

### Isolation and Identification of hUCMSC-Exos

Culture medium of hUCMSCs at the fourth to sixth passage was obtained and centrifuged at 2,000 × g for 15 min at 4°C to filter cells. Then, the supernatant was centrifuged at 10,000 g for 30 min at 4°C to filter debris after which the supernatant was centrifuged at 120,000 g for 70 min at 4°C to remove the supernatant. The precipitate was washed using phosphate-buffered saline (PBS; Servicebio, China) after which we centrifuged the supernatant at 120,000 g for 70 min at 4°C. Then, the precipitate was suspended in pre-cooled PBS (Servicebio), which led to the isolation of exosomes at a concentration of 1 mg/mL (8.69×10^10^ particles/ml). The hUCMSC-Exos were experimentally identified (26). First, we used Flow NanoAnalyzer (U30; NanoFCM, China) to measure exosomal size distribution and concentration. Then, we observed their morphologies by using transmission electron microscopy (TEM; Talos F200C; Thermo Scientific, US). Finally, surface markers of hUCMSC-Exos, including CD9, CD63, and CD81 (Biolegend, US), were detected by FCM (BD).

### Isolation and Identification of Mouse Ovarian GCs

Ethical approval for the use of animal models was obtained from the Ethical Committee of Second Hospital of Hebei Medical University. Three-week-old healthy female C57BL/6 mice were acquired from SPF Biotechnology (China). Stimulation of follicle growth was done by intraperitoneal injection of pregnant mare serum gonadotropin (Solarbio, China). Mice ovarian tissues were obtained after 48h of the injection of pregnant mare serum gonadotropin. Then, we isolated GCs under the anatomical microscope (SMZ-10A; Nikon, Japan). Next, digestion of the was conducted in 0.1% hyaluronidase (Sigma, US) at 37°C for 10 min. After terminated the digestion and washed the tissues, we filtered the suspension through a cellular strainer. After that, we cultured single-cell suspensions in DMEM containing Ham’s F12 medium (DMEM: F12; Gibco) at cellular incubation conditions. Lastly, immunofluorescence staining of follicle stimulating hormone receptors (FSHR; 1:800; Servicebio) was performed to identify GCs.

The first passage GCs were seeded on the six-well plate with a density of 1×10^5^ cells/well. We divided GCs into five groups. To confirm the protective role of exosomes against cyclophosphamide (CTX)-induced GC injury, in the CTX group, 30 μM CTX (Sigma, US) was co-cultured with GCs for 24 h. In the Exos group, we added 20 μg/mL hUCMSC-Exos and CTX (Sigma) to the cellular medium. Verteporfin (Bioss antibodies, China) was used as a YAP inhibitor that disrupts YAP-TEAD interactions. In the Exos+Verteporfin group, in addition to culturing GCs with hUCMSC-Exos and CTX, we added 10 μM verteporfin into the cellular medium. In the CTX +Verteporfin group, we added CTX and verteporfin to the medium of GCs. Lastly, in the Normal group, we cultured GCs with the common cellular medium.

### Proliferation of GCs

We conducted EdU and CCK-8 assays to assess the proliferative ability of GCs. After drug interventions and exosome-mediated treatment for all groups, we used an EdU Kit (Beyotime, China) to detect cell proliferation according to the manufacturer’s instructions. After completed the operation, we observed GCs under a fluorescence microscope (AxioCam HRc; ZEISS, Germany). The CCK-8 assay was also performed. Briefly, we seeded first passage GCs into 96-well plates with a density of 4000 cells/well. After drug interventions and exosome-mediated treatment for all groups, 10 μl of CC K-8 solution (Servicebio, China) was added into wells and incubated at cellular incubation conditions for 4 h. Absorbance was measured at 490 nm using a microplate reader (Synergy-H1; BioTek, US). The same procedure was performed after 1-5 days to evaluate GCs proliferation.

### POI Mouse Model and Treatment With hUCMSC-Exos

Eight-week-old healthy female C57BL/6 mice were acquired from SPF Biotechnology (China), and all procedures were ethically approved by the Ethics Committee of Second Hospital of Hebei Medical University. Mice were equally assigned into three groups. The POI (n=12) and Exos (n=12) groups were intraperitoneally administered with CTX (120 mg/kg) two times (Once every seven days; according to the results of our pilot study) to establish the POI model. The Normal group (n=12) received non-operate. We confirmed the establishment of POI mice models by constantly detecting vaginal smears and measuring weight for two weeks after the injection of CTX. Then, we administered mice in the Exos group by intraperitoneal transplantation of 150 μg hUCMSC-Exos two times (Once every seven days). At the same time, we administered mice in the POI group by intraperitoneal injection of 150 μl PBS. Two weeks after the first treatments (27), 18 mice (n=6 per group) were sacrificed, and blood serum and ovaries were harvested for subsequent experiments.

### Reproductive Tests

Reproductive tests were conducted to assess the efficiency of hUCMSC-Exos involved functional improvement of fertility. Two weeks after the first treatment, we mate female mice in the experimental group (n=6 per group) with sexually mature male mice at a 1:1 ratio continuing eight weeks. Fertility levels of the three groups, including pregnancy rate, number of offspring, and time of birth were recorded (28). Pregnancy rate was respectively calculated in four- and eight-week after mating. The number of offspring and the time of birth were calculated in eight-week.

### Ovarian Follicle Counts and Morphological Analysis

After the completing treatment, we obtained ovaries from sacrificed mice. Next, we fixed ovaries in 4% paraformaldehyde (Servicebio) for 48 h. After completing paraffin embedding, we sliced the ovaries into 4 μm sections. Then, we selected more than three sections of every ovary for hematoxylin-eosin (HE) staining. Lastly, we observed morphological characteristics of every ovarian section and counted all stages of follicles (primordial, primary, secondary, antral, and atretic follicles) using light microscopy (ZEISS).

### TUNEL Analysis

TUNEL analysis of ovarian sections was performed by using the Tunel Cell Apoptosis Detection Kit (Servicebio, China). Briefly, 0.3% Triton X-100 (Solarbio, China) was used for permeabilization of ovarian sections. Then, the sections were incubated with TUNEL detection solution. The DAPI solution (SouthernBiotech, US) was used to label the nucleus. Finally, sections were analyzed by fluorescence microscopy (ZEISS). The DAPI-labelled nuclei appeared blue while positive apoptosis cells appeared green.

### ELISA

After mice were sacrificed, blood was collected after which serum was obtained by centrifuging at 5000 rpm for 10 min. Anti-Mullerian hormone (AMH), estradiol (E_2_), and FSH levels in serum were measured by ELISA Kit (MEIMIAN, China) according to the manufacturer’s instructions. In brief, we added 50 μl of each serum to each coated well for incubation. After 30 min, we washed these wells five times using a wash buffer. Next, we added HRP-conjugated antibodies into the wells. After the same wash steps of wells, we added the color development solution into the wells for 10- minute incubation at 37°C. Finally, we added the stop buffer and measured the optical density (OD) at a 450 nm wavelength. The concentration of these hormones was calculated based on the standard curve.

### Immunofluorescence Staining

Paraffin-embedded mouse ovarian sections were immersed in sodium citrate buffer (Servicebio, China) at a sub-boiling temperature for 5 min, cool for 5 min at room temperature, followed by another sub-boiling temperature for 5 min for antigen retrieval. Then, 0.3% Triton X-100 was used for permeabilization after which the objective area was covered with 5% goat serum (Servicebio) for 1 h. Then threw away the blocking solution slightly and incubated the slides at 4°C overnight in the presence of primary antibodies, including FSHR (1:400; Servicebio), Ki67 (1:800; Servicebio), and proliferating cell nuclear antigen (PCNA; 1:800; Servicebio). On the next day, slides were washed with PBS (Servicebio) three times after which the sections were incubated for one hour in the presence of a secondary Alexa Fluor 488-conjugated goat anti-rabbit antibody (1:400; Servicebio, China) or Cy3 conjugated goat anti-rabbit antibody (1:400; Servicebio, China). Finally, cell nuclei were labeled with the DAPI solution (SouthernBiotech). We observed these slides by using a fluorescence microscope (ZEISS).

### Immunohistochemistry Staining

Ovaries from each group of mice were fixed, sectioned (4 μm), and incubated in the presence of primary mouse antibodies, including FSHR (1:400; Servicebio) and Ki67 (1:800; Servicebio), at 4°C overnight. Subsequently, the slides were incubated with biotinylated secondary antibodies at room temperature for one hour. Reaction products containing diaminobenzidine (ZSGB-BIO, China) were used to color the slides, which were counterstained with hematoxylin (Servicebio).

### Western Blotting Analysis

Firstly, we used the RIPA lysis buffer (Servicebio) to extract protein from mouse ovaries *in vivo* and GCs *in vitro*. Protein concentrations were measured by using a BCA protein assay kit (Solarbio, China). Then, we made protein samples denatured at 100°C for 10 min. SDS-PAGE (Servicebio) was used to separate proteins, and then proteins were transferred into 0.22 μm PVDF membranes (Sigma, US). After being blocked with 5% skim milk for 1.5 hours, membranes were incubated with primary antibodies overnight at 4°C. The next day, we washed them three times and incubated them with an HRP-conjugated goat anti-rabbit antibody (Servicebio, China) for one hour. Lastly, we detected the proteins levels using the ChemiDoc MP Imaging System (Bio-Rad, USA).

### Statistical Analysis

Each test was performed at least three times. All results were analyzed by using SPSS 26.0 software. Student’s t-test was used for comparisons between two groups. A one-way analysis of variance was used for the distribution of data. Results are presented in the form of mean ± SD. A *p* value *of* < 0.05 was considered to be significantly different.

## Results

### Characterization and Identification of hUCMSCs and hUCMSC-Exos

**Figure 1:**
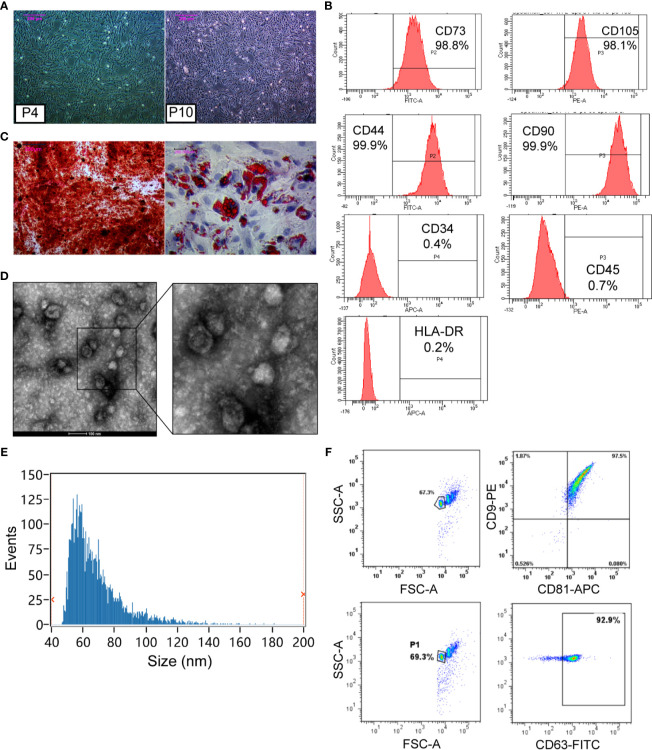
Characterization and identification of hUCMSCs and hUCMSC-Exos. **(A)** The morphology of hUCMSCs at the fourth passage and the tenth passage. Scale bar: 200 μm. **(B)** hUCMSCs were positive for CD73, CD105, CD44, and CD90, and were negative for CD34, CD45, and HLA-DR, as shown by flow cytometry analysis. **(C)** Alizarin Red staining was used for hUCMSCs osteogenic identification, and Oil Red O staining was conducted for adipogenic differentiation. Scale bar: 200 μm. **(D)** Representative images of hUCMSC-Exos under transmission electron microscopy. Scale bar: 100 nm. **(E)** Particle size distribution of hUCMSC-Exos was determined by Flow Nano Analyzer. **(F)** hUCMSC-Exos were positive for CD9, CD81, and CD63, which were shown by flow cytometry analysis.

After inoculation, the MSCs isolated from the human umbilical cord grew into long fusiform cells with fibroblast-like morphologies. In passage four to ten, cells exhibited a consistent spindle shape **(** **Figure 1A** **)**. Immunophenotypes of MSCs were characterized by flow cytometry using specific biomarkers. The results showed a high expression of CD73 (98.8%), CD105 (98.1%), CD44 (99.9%), and CD90 (99.9%), and a low expression of CD34 (0.4%), CD45 (0.7%), and HLA-DR (0.2%) in hUCMSCs **(** **Figure 1B** **)**. In addition, we induced hUCMSCs to develop into different cell types in a conditional culture system. The positive results of Alizarin Red and Oil Red O staining for induced hUCMSC demonstrated the multi-lineage differentiation potential of them **(** **Figure 1C** **)**. These characterizations meet the criteria for defining human MSC **(** **Table 1** **)** (25).

**Table 1:** Results of identification of hUCMSCs, hUCMSC-Exos and GCs, and establishment of POI model.

Identification	Methods	Results	Figure
hUCMSC	(1) Morphological Analysis	Cells exhibited a consistent spindle-shape from passage 4 to 10	**Figure 1A**
	(2) Flow cytometry	CD73, CD105, CD44, and CD90 are positive, and CD34, CD45, and HLA-DR are negative	**Figure 1B**
	(3) Osteogenic/adipogenic differentiation	Calcium deposition and neutral lipid vacuole accumulation	**Figure 1C**
Exosomes	(1) Transmission electron microscopy	Exosomes exhibited a cup-shaped morphology	**Figure 1D**
	(2) Flow NanoAnalyzer	Size distributions ranging from approximately 50 to 100 nm at a concentration of 8.69×10^10^ particles/mL	**Figure 1E**
	(3) Flow cytometry	CD9, CD81, and CD63 are positive	**Figure 1F**
Granulosa cells	(1) Morphological Analysis	Oval or polygonal shapes with a single layer	**Figure 2A**
	(2) Immunofluorescence staining	Most cells possessed FSHR	**Figure 2B**
Establishment of POI model	(1) Body weights	Body weights loss in the POI group	
	(2) Vaginal smear	Stayed in the estrous phase or lost the periodic change in the POI model	
	(3) Morphological analysis	Follicle numbers were declined, the arrangement of GCs was disordered, and interstitial fibrosis was severe in the POI group	**Figure 3B**
	(4) TUNEL analysis	More TUNEL-positive cells were observed in the POI group	**Figure 3C**

Subsequently, hUCMSC-Exos were identified according to minimal experimental requirements for definition of extracellular vesicles (26). The exosomes exhibited a cup-shaped morphology **(** **Figure 1D** **)** with their size distributions ranging from about 50 to 100 nm at a concentration of 8.69×10^10^ particles/mL **(** **Figure 1E** **)**. Additionally, the surface marker proteins were positive in exosomes by conducting FCM, including CD9, CD81, and CD63 **(** **Figure 1F** **)**. These results confirmed that exosomes had been isolated from hUCMSCs **(** **Table 1** **)**.

### Identification of GCs and the Therapeutic Effects of hUCMSC-Exos *In Vitro*

**Figure 2:**
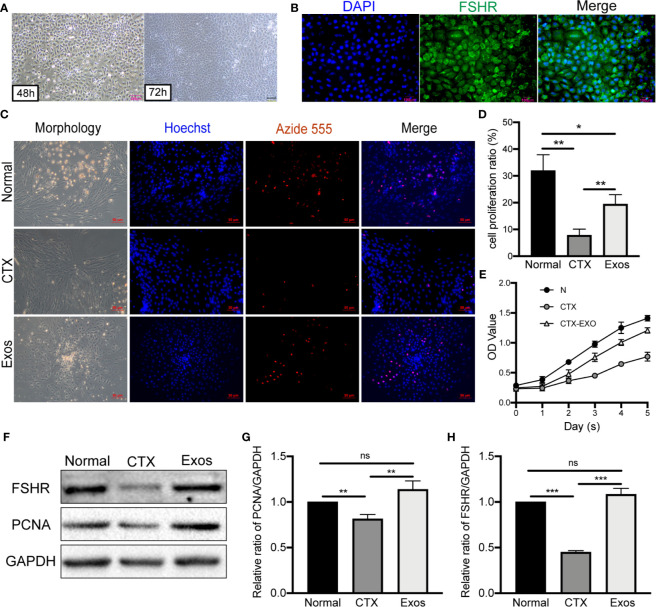
Morphology and identification of GCs and therapeutic effects of hUCMSC-Exos in CTX-damaged GCs. **(A)** Morphologies of mouse ovarian GCs after isolation at 48 h and 72 h. Scale bar: 200 μm. **(B)** Identification of GCs by FSHR immunofluorescence. Scale bar: 100 μm. **(C)** EdU assay showed hUCMSC-Exos promoted the proliferative of GCs; cell nuclei were stained blue, while cells with proliferation activity were stained red. Scale bar: 50 μm. **(D)** Proliferation ratio of GCs was analyzed through the EdU assay. **(E)** Effects of hUCMSC-Exos on GCs viability were assessed by the CCK-8 assay from day 0 to day 5. **(F)** Representative immunoblots of PCNA and FSHR, using GAPDH as the reference. Quantification of protein expression revealed that exosomal treatment increased the expression levels of **(G)** PCNA and **(H)** FSHR. Data are presented as the mean ± SD. *p < 0.05, **p < 0.01, ***p < 0.001, ns, no significance. Data are representative of three independent experiments in each group.

GCs were isolated from mouse ovaries and cultured *in vitro* to investigate the possible mechanisms of hUCMSC-Exos in POI. After 48 h of initial plating, GCs exhibited oval or polygonal shapes with a single layer. And after 72 h, cells exhibited significant proliferation under light microscopy **(** **Figure 2A** **)**. In the ovarian tissue, FSHR was a molecular marker for the identification of GCs (27). Therefore, immunofluorescence staining of FSHR was used to identify ovarian GCs. Most cells exhibited a green fluorescence, which indicated that they are GCs **(** **Figure 2B** **)**. These findings proved that ovarian GCs had been successfully isolated and cultured **(** **Table 1** **)**.

To verify the therapeutic effect of exosomes *in vitro*, they were co-cultured with CTX-damaged GCs. Briefly, proliferation of GCs was measured through EdU and CCK-8 assays. In the EdU assay, red fluorescence with Alexa Fluor 555 exhibited proliferative GCs, which were more in the Exos group compared to the CTX group **(** **Figure 2C** **)**. Moreover, the EdU assay revealed that the proliferative ratio of GCs was significantly increased in the Exos group compared with the POI group (*p* < 0.01) **(** **Figure 2D** **)**. The CCK-8 assay showed that, compared to the CTX group, the proliferation of GCs was significantly enhanced when they were co-cultured with hUCMSC-Exos for 5 days **(** **Figure 2E** **)**. Then, proteins associated with GCs proliferation and function were analyzed by western blotting. In the Exos group, proliferative proteins (PCNA) and functional proteins (FSHR) were elevated when compared to the CTX group **(** **Figures 2F–H** **)**. These findings showed that hUCMSC-Exos promote GC proliferation and expression of FSHR *in vitro*.

### hUCMSC-Exos Restored Ovarian Function in POI Mice Models

**Figure 3:**
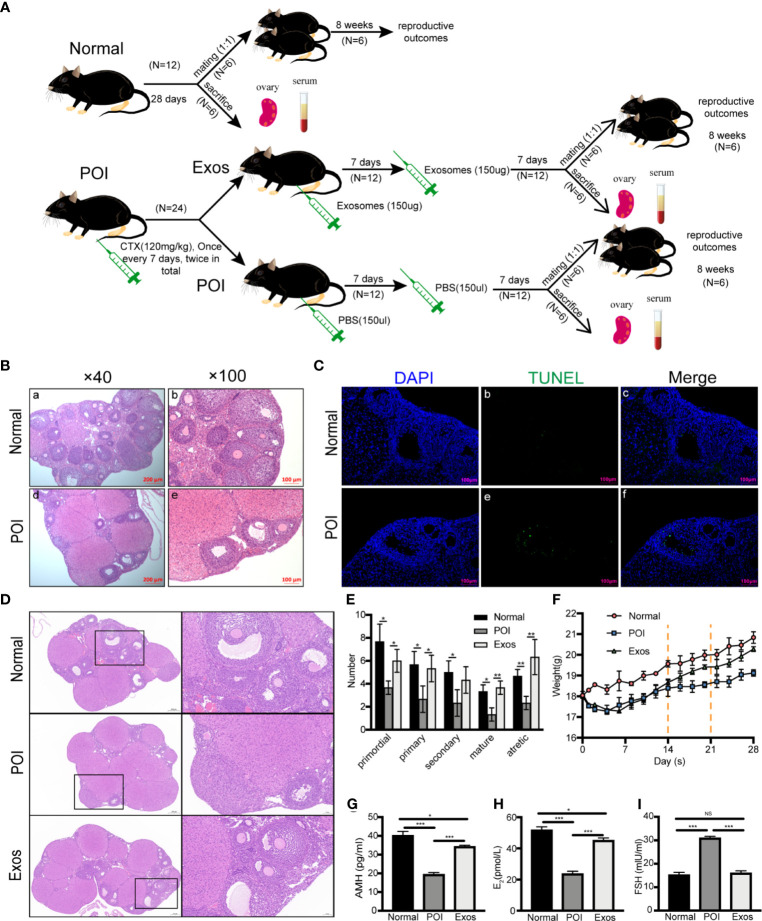
Therapeutic effects of hUCMSC-Exos in POI mice models. **(A)** Experimental timeline. After POI models were established by the injection of CTX twice, hUCMSC-Exos transplantation was performed. Seven days after the last treatment, six mice were sacrificed while six other mice were mated with male mice (1:1). **(B)** H&E staining of ovaries in the Normal and POI group. Scale bar: 100 μm and 200 μm. **(C)** Apoptosis was measured by TUNEL staining in the Normal and POI group. Cell nuclei were stained by DAPI (blue fluorescence) while apoptotic cells were stained with FITC (green fluorescence). Scale bar: 100 μm. **(D)** HE staining of ovaries in three different groups. Scale bar: 50 μm and 200 μm. **(E)** The number of follicles in different stages in each group. **(F)** Mice weight changes in the three groups. The dotted line indicates mice received treatment for 14 days and 21 days. **(G)** AMH, **(H)** E_2,_ and **(I)** FSH levels were significantly elevated in the Exo group compared to the POI group and were near normal levels. Data are presented as the mean ± SD. *p < 0.05, **p < 0.01, ***p < 0.001, ns, no significance. Data are representative of three independent experiments in each group.

Animal experiments were performed according to the experimental timeline **(** **Figure 3A** **)**. Specifically, hUCMSC-Exos transplantation we performed following the POI model was established by injecting CTX twice. Seven days after the last treatment, six mice of each group were sacrificed, and six mice were mated with male mice (1:1). To confirm the establishment of POI mice models, body weights for mice in each group were measured, and vaginal smear was performed every morning at 8 am. In the Normal group, a regular estrous cycle was four to six days. In the POI model group, almost all mice stayed in the estrous phase and have no periodic change. Compare with the Normal group, HE staining of ovarian sections revealed that follicle numbers were significantly decreased, the arrangement of GCs was disordered, and the interstitial fibrosis was more severe in the POI group **(** **Figure 3B** **)**. Furthermore, TUNEL staining was performed to detect apoptotic cells in ovarian tissues. In the POI group, there were more TUNEL-positive ovarian GCs in follicles **(** **Figure 3C** **)**. These results confirmed that the POI models had been successfully established (**Table 1**).

Then, the therapeutic effects of hUCMSC-Exos on restoration of ovarian function were analyzed. After treatment of hUCMSC-Exos, we observed gradual restoration of the estrous cycle, but not in the POI group. Compared to the POI group, HE staining of ovarian sections revealed that the numbers of follicles in all stages were significantly increased in the Exos group, and were nearly close to normal levels **(** **Figure 3D** **)**. Meanwhile, these pathological results showed that no inflammatory cells and immunological reactions appeared in the ovarian tissue after exosomes treatment, indicating low immunogenicity of MSC-derived exosomes **(** **Figure 3D** **)**. After ovaries of different groups were collected, we counted the number of follicles in each section to further analyze the therapeutic effect. Compared to the Normal group, the numbers of primordial (*p* < 0.05), primary (*p* < 0.05), secondary (*p* < 0.05), mature (*p* < 0.05), and atretic follicles (*p* < 0.01) were significantly decreased in the POI group. However, after conducting exosomal transplantation, the numbers of primordial (*p* < 0.05), primary (*p* < 0.05), mature (*p* < 0.01), and atretic follicles (*p* < 0.01) were significantly increased than they were in the POI group **(** **Figure 3E** **)**. Taken together, the number of different stages of follicles in the Exos group exhibited an increasing trend compared to the POI group. Before CTX injection, the body weights of mice were almost at the same levels among the three groups. After injection of CTX, mice in the Exos and POI groups lost weight gradually within seven days compared to them in the Normal group. However, After the first hUCMSC-Exos transplantation, body weights of mice in the Exos group exhibited a gradual increase following the first exosomal transplantation, while mice in the POI group only exhibited a slow weight gain **(** **Figure 3F** **)**. Then, we measured AMH, E_2_, and FSH levels in the serum of POI models. Compared with the Normal group, the AMH (*p* < 0.001) **(** **Figure 3G** **)** and E_2_ (*p* < 0.001) **(** **Figure 3H** **)** levels were significantly decreased, and FSH (*p* < 0.001) **(** **Figure 3I**) levels were remarkably elevated in the POI group. The change of hormones levels implying the successful establishment of POI mice models. After exosomal administration, AMH (*p* < 0.001) **(** **Figure 3G** **)** and E_2_ (*p* < 0.001) **(** **Figure 3H** **)** levels were significantly increased, while FSH (*p* < 0.001) **(** **Figure 3I** **)** levels were notably suppressed when compared to the POI group. However, AMH (*p* < 0.05) and E_2_ (*p* < 0.05) levels in the Exos group had not returned to normal levels after two weeks of treatment when compared to the normal group. These findings showed that exosomal transplantation promoted the recovery of ovarian functions and physiological functions of POI mice.

### hUCMSC-Exos Improved Reproductive Ability of POI Mice

**Figure 4:**
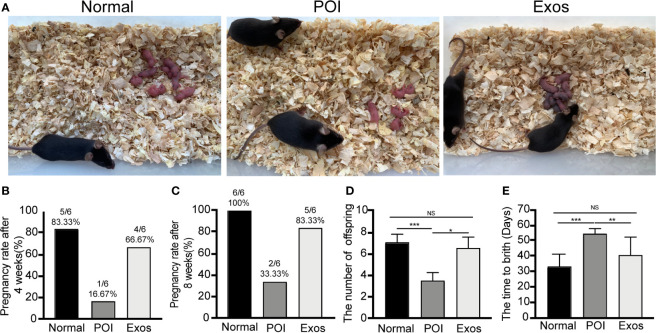
Exosomes improved reproductive functions of POI mice. **(A)** Representative outcomes of reproduction in the three groups. **(B)** Pregnancy rate at 4 weeks after treatment. **(C)** Cumulative pregnancy rate at 8 weeks after treatment. **(D)** The number of offspring in the three groups. **(E)** Time to birth after treatment. Data are presented as percentage or mean ± SD; n = 6 per group; *p < 0.05, **p < 0.01, ***p < 0.001; ns, no significance.

Fertility results of the three groups, including pregnancy rate, number of offspring, and time to birth, were recorded. Representative figures of fertility results showed that the transplantation of exosomes significantly improved the reproductive functions of POI mice **(** **Figure 4A** **)**. Four weeks after exosomal transplantation, pregnancy rate of mice in the Exos group was higher (66.67%) when compared to that in the POI group (16.67%) **(** **Figure 4B** **)**. In eight weeks, the rate was 83.33% and 33.33%, respectively **(** **Figure 4C** **)**. Moreover, the number of offspring was significantly suppressed in the POI group compared with the Normal group (*p* < 0.001). In contrast, treatment with hUCMSC-Exos significantly increased the number of offspring compared with the POI group (*p* < 0.05) **(** **Figure 4D** **)**. Additionally, time to birth in the POI group was significantly prolonged (*p* < 0.001), which was reduced by exosomal transplantation (*p* < 0.01) **(** **Figure 4E** **)**. These findings showed that exosomal administering could greatly improve reproductive ability of POI mice.

### hUCMSC-Exos Restored GCs FSHR Expression and Promoted Proliferation of GCs *In Vivo* Through the Hippo Pathway

**Figure 5:**
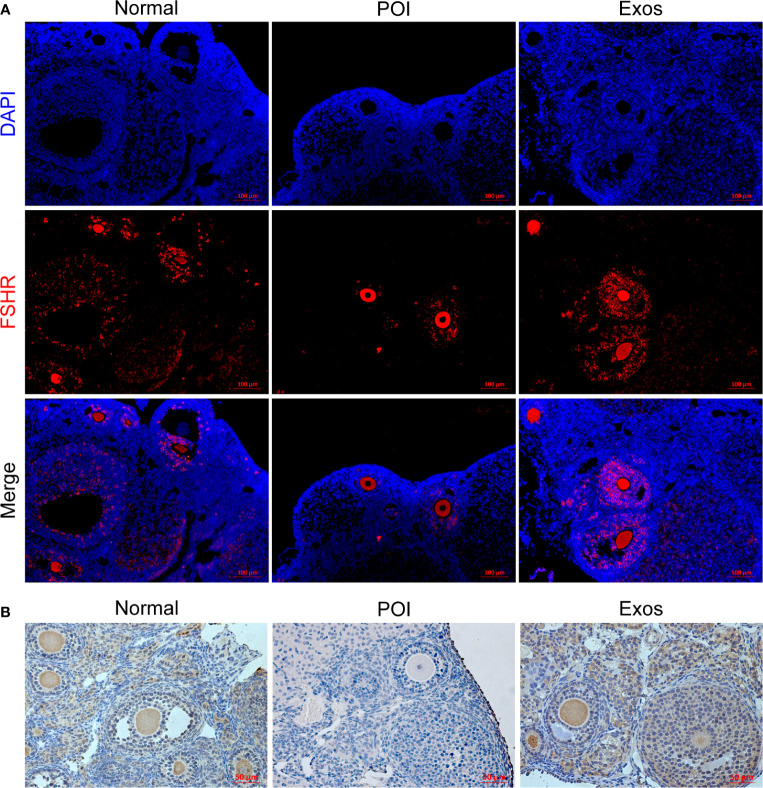
Exosomes restored the expression levels of FSHR in GCs *in vivo*. **(A)** Expression levels of FSHR in ovaries as measured by immunofluorescence staining. Scale bar: 100 μm. **(B)** Expression levels of FSHR in ovaries as measured by immunohistochemical staining. Scale bar: 50 μm.

FSHR is a crucial molecular for GCs to perform their roles in regulating follicular function and activity. Therefore, immunological methods for ovarian sections were used to detect the expressions of FSHR in each group. Immunofluorescence staining of FSHR showed that more functional follicular GCs were observed in the Exos and Normal groups when compared to the POI group **(** **Figure 5A** **)**. Furthermore, immunohistochemical staining revealed the same results **(** **Figure 5B** **).** High expression levels of FSHR indicated that hUCMSC-Exos transplantation improved folliculogenesis and follicular function.

**Figure 6:**
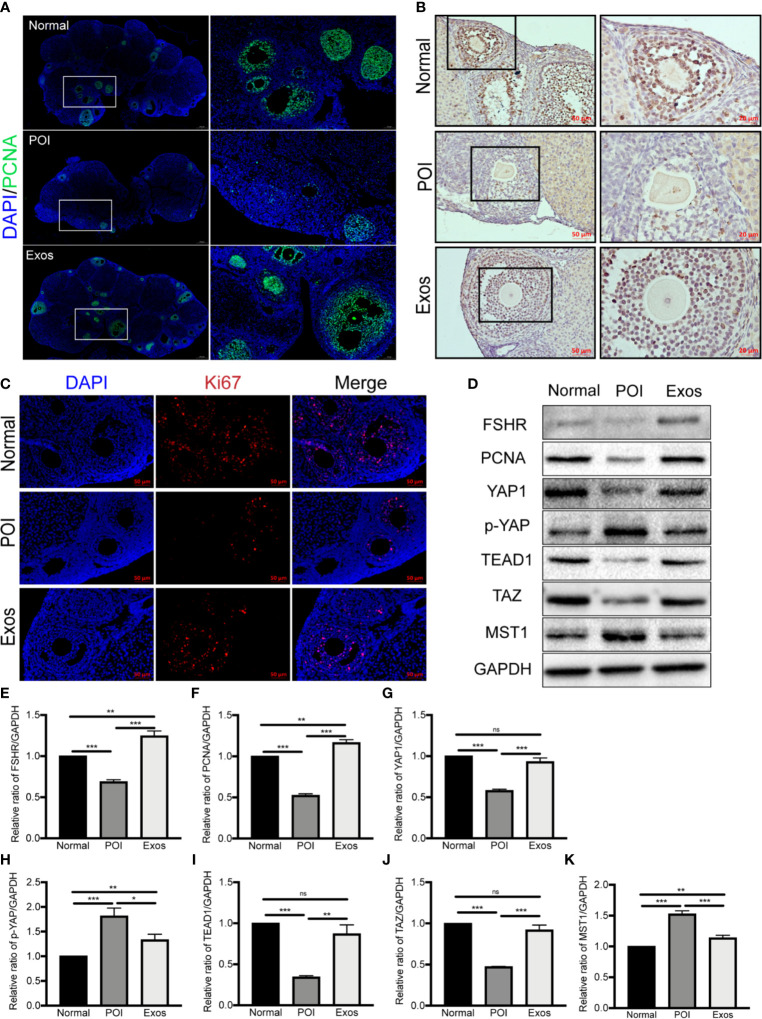
Exosomes promoted ovarian proliferation by regulating the Hippo pathway in POI mice. **(A)** Expression of PCNA was measured by immunofluorescence staining in the different groups. Scale bar: 50 μm and 200 μm. Expression of Ki67 was measured by **(B)** immunohistochemical staining and **(C)** immunofluorescence staining in the three groups. Scale bar: 20 μm and 50 μm. **(D)** Representative immunoblots of biomarkers of proliferation and the Hippo pathway, using GAPDH as reference. Quantification of protein expression revealed that exosomal treatment elevated the expression levels of **(E)** FSHR, **(F)** PCNA, **(G)** YAP1, **(I)** TEAD1, and **(J)** TAZ, and suppressed the expression of **(H)** p-YAP and **(K)** MST1. Data are presented as mean ± SD. *p < 0.05, **p < 0.01, ***p < 0.001, ns, no significance. Data are representative of three independent experiments in each group.

PCNA and ki67 are the most conventional molecular markers for detecting proliferative cells. Accordingly, the detection of them was performed to evaluate the efficiency of exosomes in promoting ovarian cells proliferation. Immunofluorescence staining of PCNA showed more follicular proliferative GCs in the Exos and Normal group compared with the POI group **(** **Figure 6A** **)**. Similarly, Immunohistochemical **(** **Figure 6B** **)** and immunofluorescence staining **(** **Figure 6C** **)** of Ki67 indicated that there were more proliferative cells in the Exos than in the POI group. Therefore, treatment with hUCMSC-Exos promoted ovarian cells proliferation, which contributed to the restoration of follicular and ovarian functions.

The Hippo signaling pathway plays a vital role in folliculogenesis and activation of primordial follicles (21). To determine the regulatory role of Hippo pathway in the hUCMSC-Exos-mediated therapeutic effects on POI mice models, proteins associated with key Hippo molecules and ovarian function (FSHR and PCNA) were simultaneously detected by western blotting. In the Exos group, proteins expression levels of YAP1, TAZ, and TEAD1, and ovarian functional proteins (FSHR and PCNA) were significantly elevated compared to the POI group. Conversely, proteins levels of p-YAP and MST1 were suppressed in the Exos group **(** **Figures 6D–K** **)**. These findings preliminary indicate that hUCMSC-Exos promoted ovarian proliferation and function by regulating the Hippo pathway.

### hUCMSC-Exos Promoted GCs Proliferation *In Vitro* by Regulating the Hippo Pathway and the Effect Was Inhibited by a YAP Inhibitor

**Figure 7:**
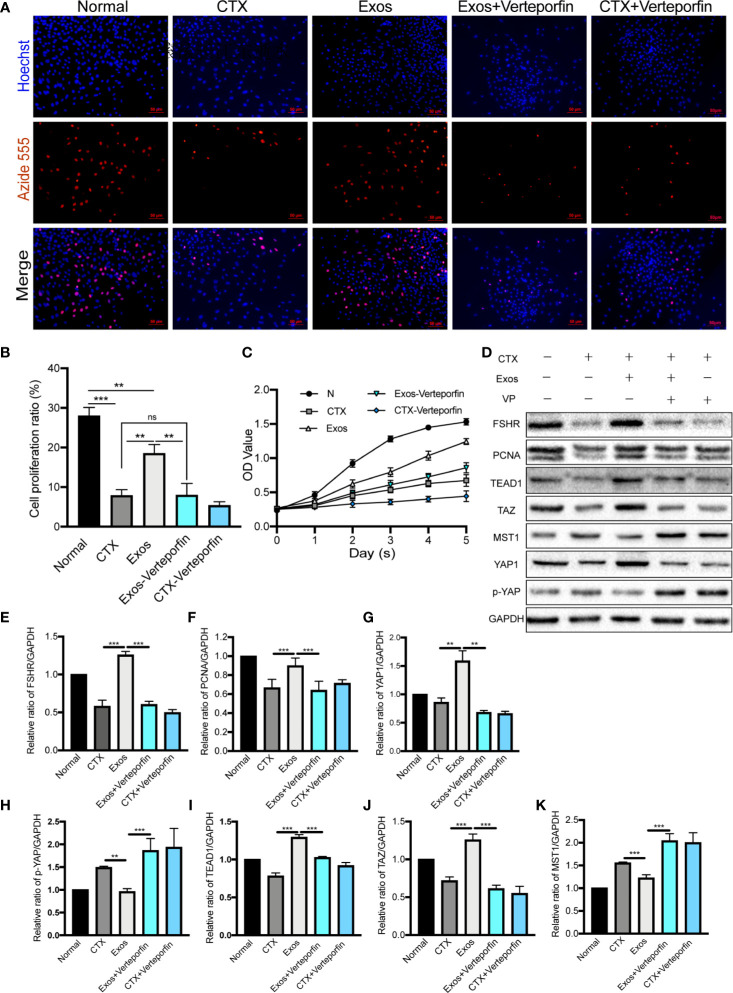
Exosomes promoted GCs proliferation by regulating the Hippo pathway *in vitro*. **(A)** The EdU assay showed that hUCMSC-Exos promoted GCs proliferation, while the inhibitor of YAP, verteporfin, reversed this effect. Scale bar: 50 μm. **(B)** Proliferative ratio of GCs in five different groups was analyzed through the EdU assay. **(C)** Cell proliferation curve of GCs from day 0 to day 5 in the five groups, as measured by the CCK-8 assay. **(D)** Representative immunoblots of biomarkers of proliferation and the Hippo pathway in the five groups, using GAPDH as reference. Quantification of protein expression levels of **(E)** FSHR, **(F)** PCNA, **(G)** YAP1, **(I)** TEAD1, **(J)** TAZ, **(H)** p-YAP, and **(K)** MST1 revealed that exosomes promoted GCs proliferation by regulating the Hippo pathway, and the therapeutic effects were suppressed by verteporfin. Data are presented as the mean ± SD. *p < 0.05, **p < 0.01, ***p < 0.001, ns, no significance. Data are representative of three independent experiments in each group.

Verteporfin was used as an inhibitor of YAP in cultured GCs with/without hUCMSC-Exos to determine whether the pathway is involved in GCs proliferation and function *in vitro*. The proliferative ability of GCs was evaluated in different groups by using EdU and CCK-8 assays. In EdU assay, the effect of hUCMSC-Exos on promoting GCs proliferation was disappeared after co-culture with verteporfin **(** **Figure 7A** **)**. Additionally, the proliferative rate was significantly increased in the Exos group compared with the CTX group (Exos group *vs* CTX group; *p* < 0.01), and the rate was notably decreased when verteporfin was added to the cell culture medium (Exos group *vs* Exos-Verteporfin group; *p* < 0.01) **(** **Figure 7B** **)**. In the CCK-8 assay **(** **Figure 7C** **)**, similar results were observed. These findings suggest that proliferative ability of GCs was significantly enhanced by co-culture with hUCMSC-Exos after CTX pretreated, but this therapeutic effect disappeared following the addition of a YAP inhibitor.

Furthermore, proteins associated with the Hippo pathway, and proliferation (PCNA) and function (FSHR) of GCs, were detected by Western blot analysis **(** **Figure 7D** **)**. After co-cultured with hUCMSC-Exos, expression levels of proteins of YAP1 (*p* < 0.01), TAZ (*p* < 0.001), TEAD1 (*p* < 0.001), PCNA (*p* < 0.001), and FSHR (*p* < 0.001) in GCs were significantly elevated compared to the CTX group **(** **Figures 7E–G, I, J** **)**. Meanwhile, proteins levels of p-YAP (*p* < 0.01) and MST1 (*p* < 0.001) were decreased **(** **Figures 7H, K** **)**. However, contrasting results were found when the YAP inhibitor was added to the medium (Exos group *vs* Exos-Verteporfin group; YAP1, *p* < 0.01; TAZ, *p* < 0.001; TEAD1, *p* < 0.001; PCNA, *p* < 0.001; FSHR, *p* < 0.001; p-YAP, *p* < 0.001; MST1, *p* < 0.001) **(** **Figures 7D–K** **)**.

**Figure 8:**
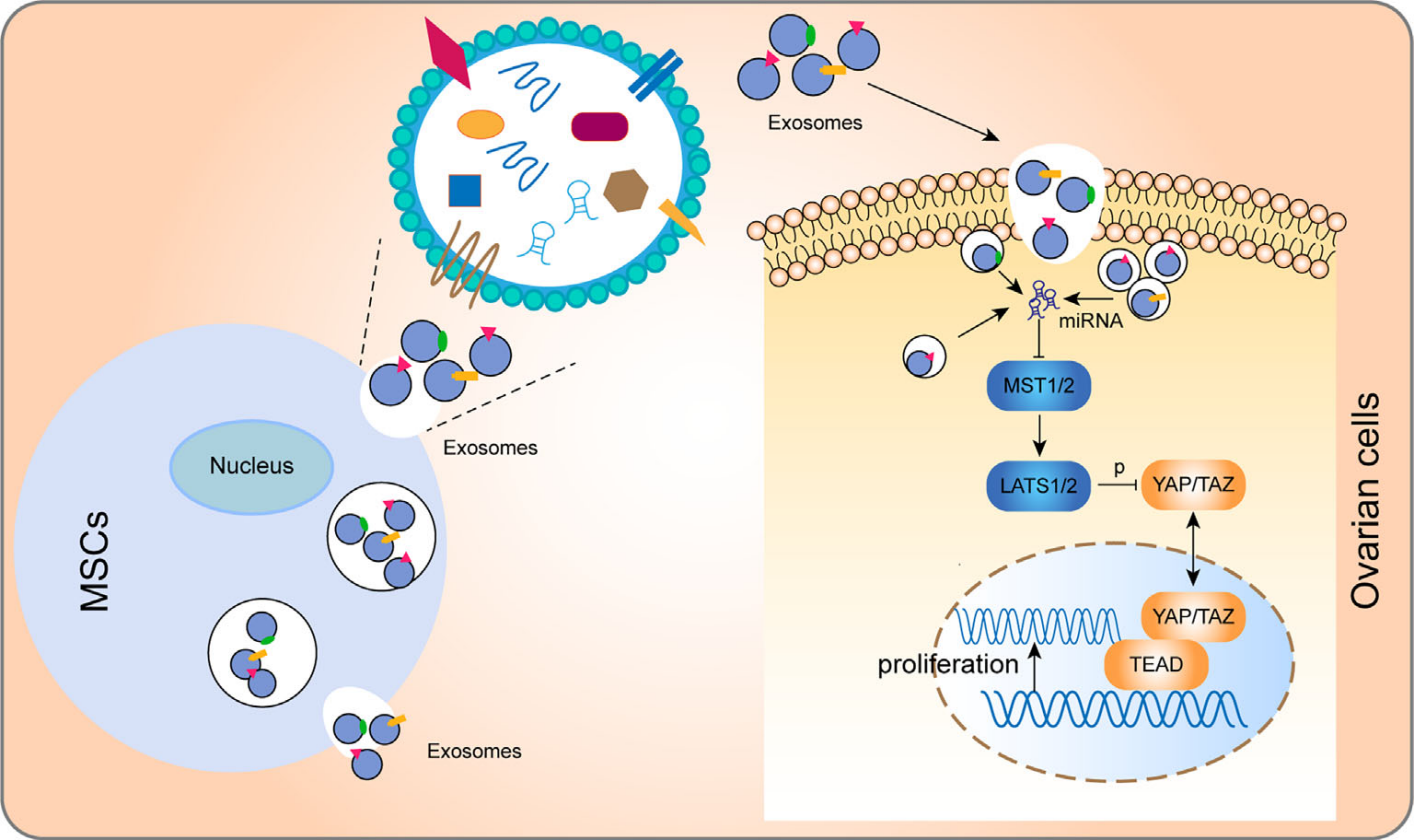
Model for how hUCMSC-Exos increase ovarian cells proliferation by regulating the Hippo pathway. In brief, after phagocytosis by ovarian cells, exosomes regulated key molecules of the Hippo through the non-coding RNA and other molecules that they convey to cells, thereby regulating the Hippo pathway and promoting cell proliferation.

In summary, these findings show that hUCMSC-derived exosomes promote the proliferation and function of ovarian cells by regulating the Hippo pathway **(** **Figure 8** and **Table 2** **)**. These therapeutic effects were suppressed when the key Hippo molecule (YAP) was blocked, further confirming the necessity and indispensability of the Hippo pathway in exosome-mediated treatment for POI.

**Table 2:** Results of hUCMSC-Exos treatment.

	Methods	Results	*P* value
Exosomal treatment *in vivo*	(1) Vaginal smear	Estrous cycle was gradually restored to normal after treatment	
	(2) Morphological analysis	Follicles were significantly increased in the Exos group (**Figure 3D**)	
	(3) Ovarian follicle counts	Numbers of primordial, primary, secondary, mature, and atretic follicles were significantly increased following exosomal transplantation (**Figure 3E**)	Primordial: POI *vs* Normal, *P* < 0.05; POI *vs* Exos, *P* < 0.05. Primary: POI *vs* Normal, *P* < 0.05; POI *vs* Exos, *P* < 0.05. Secondary: POI *vs* Normal, *P* < 0.05; POI *vs* Exos, *P >0.05*. Mature: POI *vs* Normal, *P* < 0.05; POI *vs* Exos, *P* < 0.01. Atretic: POI *vs* Normal, *P* < 0.01; POI *vs* Exos, *P* < 0.01.
	(4) Body weights	Body weights in the Exos group exhibited a gradual increase (**Figure 3F**)	
	(5) Hormones levels in serum	AMH and E_2_ were significantly elevated, while FSH levels were suppressed after exosomal administration (**Figure 3G-I**)	AMH: POI *vs* Normal, *P* < 0.001; POI *vs* Exos, *P* < 0.001; Exos *vs* Normal, *P* < 0.05. E_2_: POI *vs* Normal, *P* < 0.001; POI *vs* Exos, *P* < 0.001; Exos *vs* Normal, *P* < 0.05. FSH: POI *vs* Normal, *P* < 0.001; POI *vs* Exos, *P* < 0.001; Exos *vs* Normal, *P* > 0.05.
	(6) Reproductive tests	Exosomes significantly improved reproductive functions of POI mice model (**Figures 4A–E**)	Number of offspring: POI *vs* Normal, *P* < 0.001; POI *vs* Exos, *P* < 0.05; Exos *vs* Normal, *P* > 0.05. Time of birth: POI *vs* Normal, *P* < 0.001; POI *vs* Exos, *P* < 0.01; Exos *vs* Normal, *P* > 0.05.
	(7) FSHR expression in ovaries	I Immunofluorescence and Immunohistochemistry staining of FSHR revealed that more functional GCs were observed in the Exos group (**Figures 5A, B**)	
	(8) Proliferative ability	Immunofluorescence staining of PCNA and Ki67, Immunohistochemistry staining of Ki67 showed hUCMSC-Exos promoted ovarian proliferation (**Figures 6A–C**)	
	(9) Mechanism exploration	hUCMSC-Exos promoted ovarian proliferation by regulating the Hippo pathway (**Figures 6D–K**)	FSHR: POI *vs* Normal, *P* < 0.001; POI *vs* Exos, *P* < 0.001; Exos *vs* Normal, *P* < 0.01. PCNA: POI *vs* Normal, *P* < 0.001; POI *vs* Exos, *P* < 0.001; Exos *vs* Normal, *P* < 0.01. YAP1: POI *vs* Normal, *P* < 0.001; POI *vs* Exos, *P* < 0.001; Exos *vs* Normal, *P* > 0.05. p-YAP: POI *vs* Normal, *P* < 0.001; POI *vs* Exos, *P* < 0.05; Exos *vs* Normal, *P* < 0.01. TEAD1: POI *vs* Normal, *P* < 0.001; POI *vs* Exos, *P* < 0.01; Exos *vs* Normal, *P* > 0.05. TAZ: POI *vs* Normal, *P* < 0.001; POI *vs* Exos, *P* < 0.001; Exos *vs* Normal, *P* > 0.05. MST1: POI *vs* Normal, *P* < 0.001; POI *vs* Exos, *P* < 0.001; Exos *vs* Normal, *P* < 0.01.
Exosomal treatment *in vitro*	(1) EdU assay	Proliferation of GCs was significantly increased in the Exos group (**Figures 2C, D**)	Cell proliferation ratio: CTX *vs* Normal, *P* < 0.01; CTX *vs* Exos, *P* < 0.01; Exos *vs* Normal, *P* < 0.05.
	(2) CCK-8 assay	Proliferation of GCs was significantly enhanced in the Exos group (**Figure 2E**)	
	(3) Western blot	PCNA and FSHR were elevated in the Exos group (**Figures 2F–H**)	PCNA: CTX *vs* Normal, *P* < 0.01; CTX *vs* Exos, *P* < 0.01; Exos *vs* Normal, *P* > 0.05. FSHR: CTX *vs* Normal, *P* < 0.001; CTX *vs* Exos, *P* < 0.001; Exos *vs* Normal, *P* > 0.05.
Therapeutic Mechanism	(1) EdU assay	hUCMSC-Exos promoted GC proliferation *in vitro* by regulating the Hippo pathway and the effect was inhibited by a YAP inhibitor (**Figures 7A, B**)	Cell proliferation ratio: CTX *vs* Normal, *P* < 0.001; CTX *vs* Exos, *P* < 0.01; Exos *vs* Normal, *P* < 0.01; Exos-Verteporfin *vs* Exos, *P* < 0.01; Exos-Verteporfin *vs* CTX, *P >*0.05.
	(2) CCK-8 assay	hUCMSC-Exos promoted GC proliferation and the effect was inhibited by a YAP inhibitor (**Figure 7C**)	
	(3) Western blot	hUCMSC-Exos elevated GCs proliferation and function by regulating the Hippo pathway (**Figures 7D–K**)	FSHR: CTX *vs* Exos, *P* < 0.001; Exos *vs* Exos-Verteporfin, *P* < 0.001. PCNA: CTX *vs* Exos, *P* < 0.001; Exos *vs* Exos-Verteporfin, *P* < 0.001. YAP1: CTX *vs* Exos, *P* < 0.01; Exos *vs* Exos-Verteporfin, *P* < 0.01. p-YAP: CTX *vs* Exos, *P* < 0.01; Exos *vs* Exos-Verteporfin, *P* < 0.001. TEAD1: CTX *vs* Exos, *P* < 0.001; Exos *vs* Exos-Verteporfin, *P* < 0.001. TAZ: CTX *vs* Exos, *P* < 0.001; Exos *vs* Exos-Verteporfin, *P* < 0.001. MST1: CTX *vs* Exos, *P* < 0.001; Exos *vs* Exos-Verteporfin, *P* < 0.001.

## Discussion

In this study, we found that hUCMSC-Exos significantly improved ovarian function and reproductive ability of POI mice models by promoting proliferation through the Hippo pathway. To confirm the effects of hUCMSC-Exos in POI, we performed well-designed experiments *in vivo* and *in vitro*. Exosome-mediated transplantation *in vivo* restored related hormone levels to nearly normal, increased the number of follicles, and promoted ovarian cell proliferation, especially the GCs. These therapeutic effects for POI models were accomplished by regulating the Hippo pathway. To further determine the therapeutic mechanism, we conducted a systematic GCs-related investigation *in vitro.* Briefly, *in vitro* co-cultured with exosomes elevated proliferation and function of GCs by regulating Hippo pathway. However, these therapeutic effects were notably reversed by the inhibitor of YAP. This study is fundamental for further clinical conduction of this novel approach for POI. Additionally, several previous studies have confirmed the therapeutic effects of MSC-derived EVs in POI models (27, 29, 30). These investigations and our findings together show bright prospects of exosome-related treatment for administering POI patients.

Ovary is the most important reproductive organ for a woman. A normal healthy ovary can perform the production of sex hormones and gametogenic functions (31). In POI, ovarian physiological functions and reproductive abilities are injured by the dysfunction or depletion of ovarian follicles. GCs and oocytes are the components of follicles. Importantly, GCs play a critical role in follicular evolution, activation, and function (32). Hormone receptors of GCs, including estrogen receptor and FSHR, are crucial for folliculogenesis and ovulation. Excessive apoptosis of GCs is a key mechanism for follicular atresia (33), which can trigger follicle dysfunction and physiological change of ovaries. It suggests that promoting proliferation of GCs can rescue damaged ovarian structures and functions. Furthermore, exploring the therapeutic effect of hUCMSC-Exos in GCs could help to determine the underlying mechanism of exosome-related treatment in POI. Therefore, hUCMSC-Exos and GCs were successfully isolated and identified before conducting exosomal treatment **(** **Figures 1**, **2** **)**. *In vitro*, co-cultured with hUCMSC-Exos significantly improved the proliferation levels of CTX-damaged GCs **(** **Figure 2** **)**. *In vivo*, excessive apoptosis of GCs and numerous atretic follicles were detected in POI mice models. After performed exosome-related transplantation, promoted ovarian cells proliferation and restored ovarian function were observed **(** **Figures 3**, **5**, **6** **)**. Briefly, all stages of follicles and proliferative ovarian cells were significantly increased after injection of hUCMSC-Exos, indicating that exosomes could improve the proliferation of GCs and folliculogenesis.

Infertility is a serious physiological impact on POI patients. Therefore, promoting fertility of females is the central goal of POI treatment. To verify the efficiency of hUCMSC-Exos treatment, we mated male and female mice in each group for eight weeks after exosomal transplantation and observed the fertility outcome. It showed that the reproductive functions of POI mice were significantly improved after exosome-mediated treatment **(** **Figure 4** **)**. Briefly, the pregnancy rate of mice and the number of offspring were enhanced, and the time to birth was shortened following the transplantation of hUCMSC-Exos. However, it is necessary to enlarge the animal numbers in further study, and more complete results should be confirmed in pre-clinical or clinical trials.

Elucidation of the mechanism involved in exosomal-mediated treatment can guarantee effective and targeted application of exosomes. The Hippo signaling pathway was originally determined in *Drosophila melanogaster* as an essential regulator of various biological processes and tissue growth, including cell growth, organ size control, and folliculogenesis (21, 34, 35). In the Hippo, MST1/2 phosphorylate LATS1/2, and then LATS1/2 directly phosphorylate the YAP and TAZ, thereby inhibiting their nuclear localization. When the Hippo pathway is functioning, YAP/TAZ are dephosphorylated and accumulate in the nucleus, where they bind with TEAD to induce gene transcription (34) **(** **Figure 8** **)**. In folliculogenesis, the Hippo pathway plays a vital role by regulating primordial follicular development and GCs proliferation. Elevated YAP, TEAD, and TAZ levels as well as suppressed MST1 and LATS1/2 levels can promote follicular development of the ovary (22). Therefore, regulating Hippo pathway is a potential therapeutic mechanism for exosome-related treatment to improve the recovery of ovarian function, the activation of GCs proliferation, and the stimulation of follicular growth. In this study, proteins that represent the activation and stimulation of follicular development (YAP, TAZ, and TEAD), and proliferation (PCNA) and function (FSHR) of GCs were suppressed in the POI group, but significantly elevated after hUCMSC-Exos treatment **(** **Figure 6** **)**. Furthermore, we designed supplementary experiments *in vitro* using verteporfin as an inhibitor of YAP, and found that the therapeutic effect of exosomes on CTX-damaged GCs was inhibited **(** **Figure 7** **)**. These findings all together prove that hUCMSC-Exos promote ovarian cells proliferation and restore ovarian functions through the Hippo signaling pathway. However, the specific mechanism of how hUCMSC-Exos activate the Hippo pathway is still unclear and needs further exploration in our further study.

The immunological reaction of exosomal transplantation crossing species is an important issue that should be concerned. Numerous investigations have demonstrated the low immunogenicity of MSC-derived exosomes in administering various diseases (36). Meanwhile, MSC-derived exosomes have similar hypoimmunogenic properties. There was no major histocompatibility complex I or II has been detected by using comprehensive proteomic analysis (37). More than 65% of *in vivo* studies have transplanted exosomes derived from human MSCs into different species of animal models (36). Inspiringly, there was no definite immunogenicity has been shown in these species crossing research. Consequently, MSC-derived exosomes are considered hypoimmunogenic. Pathological results in this study also show that there is no inflammatory response and no inflammatory cells infiltration in the ovarian tissue following the species crossing transplantation of MSC-derived exosomes **(** **Figure 3D** **)**. However, further studies are needed to confirm that the hypo-immunogenicity properties of MSC-derived exosomes are consistent among enough experimental individuals and different diseases.

Over 300 clinical trials involving MSC therapies have been completed, and some results have shown MSCs exhibit therapeutic effects in various disorders. Meanwhile, 15 clinical trials of EVs have been registered in ClinicalTrial.gov (38). If proved to be effective, MSC- and EV-based therapies will be available for clinical applications in the future. However, various challenges are associated with practical applications of MSCs and exosomes. First, there is a need to verify the safety of stem cell- and exosome-based transplantation. Second, different studies proposed distinctive strategies for isolation, culture, and identification of MSCs and MSC-derived exosomes for treatment (39), which leads to different outcomes in various experiments and clinical trials. Therefore, there is a need to develop a systematic standard for them from culture to application (40). Importantly, principles and strategies of applying MSCs and MSC-derived exosomes should coincide with their underlying treatment mechanisms. Therefore, mechanistic studies of MSCs or MSC-derived exosomes in different diseases may guide establishing a systematic standard for their clinical applications (41).

Although promising therapeutic efficiencies of MSC-derived exosomes in POI were showed, our study has certain limitations. First, the contents of hUCMSC-Exos were not evaluated. We did not investigate specific regulatory mechanisms of hUCMSC-Exos in POI. In our follow-up study, we will perform miRNA sequencing of hUCMSC-Exos to find specific miRNAs, which can regulate key Hippo molecules. Studies on the contents of hUCMSC-Exos are beneficial to elucidate specific therapeutic mechanisms. Second, we did not label the implanted hUCMSC-Exos as there were concerns that labels might alter the bioactivities of exosomes. Third, the route of administration, dosage of exosomes, and other standards should be further evaluated. Then, we did not detect inflammatory factors in the serum and ovarian tissue to evaluate the side effect of immunological reactions following exosomal treatment in this species crossing research. In the following study, more immune indicators in serum and ovarian tissue will be detected to confirm the hypo-immunogenicity properties of MSC-derived exosomes. Last but not least, as a preliminary study to verify the therapeutic efficiency of exosomes in POI mice models, the number of animals in this study is not enough to provide enough evidence for clinical transplantation of exosomes. Based on the findings of this study, we are conducting a thorough and systematic investigation. Enough animals have been included to observe the therapeutic effects of MSC-derived exosomes in POI at different times and to obtain comprehensive understandings of the underlying mechanism.

## Conclusions

In conclusion, our findings demonstrated that transplantation of hUCMSC-Exos could restore the ovarian function of POI by promoting proliferation through the Hippo signaling pathway. This study elucidates the underlying mechanism involved exosome-mediated treatment of POI. Therefore, it will become fundamental for further clinical conduction of MSC- or exosome-related therapies for POI patients.

## Data Availability Statement

The raw data supporting the conclusions of this article will be made available by the authors, without undue reservation.

## Ethics Statement

The animal study was reviewed and approved by Institution Animal Ethics Committee of the Second Hospital of Hebei Medical University.

## Author Contributions

ZL, JKZ, and XH were responsible for the concept of the research. ZL performed the *in vivo* and *in vitro* experiments and drafted the manuscript. HZ and YT contributed to the culture of hUCMSCs and performed the isolating and identification of hUCMSCs and hUCMSC-Exos. MZ and QL participated in data analysis and made the figures in this manuscript. JHZ, YPT, and MZ participated in the discussion and revised the manuscript. XH and ZL were responsible for the critical review of the manuscript. All authors contributed to the article and approved the submitted version.

## Conflict of Interest

Authors HZ and YT were employed by the company Qilu Cell Therapy Technology Co., Ltd.

The remaining authors declare that the research was conducted in the absence of any commercial or financial relationships that could be construed as a potential conflict of interest.

## Publisher’s Note

All claims expressed in this article are solely those of the authors and do not necessarily represent those of their affiliated organizations, or those of the publisher, the editors and the reviewers. Any product that may be evaluated in this article, or claim that may be made by its manufacturer, is not guaranteed or endorsed by the publisher.
